# TRPM7 restrains plasmin activity and promotes transforming growth factor-β1 signaling in primary human lung fibroblasts

**DOI:** 10.1007/s00204-022-03342-x

**Published:** 2022-07-21

**Authors:** Sarah Zeitlmayr, Susanna Zierler, Claudia A. Staab-Weijnitz, Alexander Dietrich, Fabienne Geiger, F. David Horgen, Thomas Gudermann, Andreas Breit

**Affiliations:** 1 Walther Straub Institute of Pharmacology and Toxicology, Medical Faculty, LMU Munich, Goethestrasse 33, 80336 Munich, Germany; 2 Faculty of Medicine, Johannes Kepler University, Life Science Park, Huemerstraße 3-5, 4020 Linz, Austria; 3 Institute of Lung Health and Immunity and Comprehensive Pneumology Center, Helmholtz Zentrum München GmbH, Member of the German Center for Lung Research, Max-Lebsche-Platz 31, 81377 Munich, Germany; 4 Department of Natural Sciences, Hawaii Pacific University, Kaneohe, HI 96744 USA

**Keywords:** Pulmonary fibrosis, Primary human lung fibroblasts, TGF-β1, TRPM7, Plasmin

## Abstract

Sustained exposure of the lung to various environmental or occupational toxins may eventually lead to pulmonary fibrosis, a devastating disease with no cure. Pulmonary fibrosis is characterized by excessive deposition of extracellular matrix (ECM) proteins such as fibronectin and collagens. The peptidase plasmin degrades the ECM, but protein levels of the plasmin activator inhibitor-1 (PAI-1) are increased in fibrotic lung tissue, thereby dampening plasmin activity. Transforming growth factor-β1 (TGF-β1)-induced activation of SMAD transcription factors promotes ECM deposition by enhancing collagen, fibronectin and PAI-1 levels in pulmonary fibroblasts. Hence, counteracting TGF-β1-induced signaling is a promising approach for the therapy of pulmonary fibrosis. Transient receptor potential cation channel subfamily M Member 7 (TRPM7) supports TGF-β1-promoted SMAD signaling in T-lymphocytes and the progression of fibrosis in kidney and heart. Thus, we investigated possible effects of TRPM7 on plasmin activity, ECM levels and TGF-β1 signaling in primary human pulmonary fibroblasts (pHPF). We found that two structurally unrelated TRPM7 blockers enhanced plasmin activity and reduced fibronectin or PAI-1 protein levels in pHPF under basal conditions. Further, TRPM7 blockade strongly inhibited fibronectin and collagen deposition induced by sustained TGF-β1 stimulation. In line with these data, inhibition of TRPM7 activity diminished TGF-β1-triggered phosphorylation of SMAD-2, SMAD-3/4-dependent reporter activation and PAI-1 mRNA levels. Overall, we uncover TRPM7 as a novel supporter of TGF-β1 signaling in pHPF and propose TRPM7 blockers as new candidates to control excessive ECM levels under pathophysiological conditions conducive to pulmonary fibrosis.

**Supplementary Information:**

The online version contains supplementary material available at 10.1007/s00204-022-03342-x.

## Introduction

Pulmonary fibrosis caused by chronic lung injury is characterized by affluent deposition of ECM leading to stiffness of lung tissue and reduced gas exchange (Kristensen et al. 2014). The prevalence per 10,000 individuals of the population ranges from 0.57 to 4.51 in Asia–Pacific countries, 0.33 to 2.51 in Europe, and 2.40 to 2.98 in North America (Maher et al. 2021). As no effective therapy apart from lung transplantation is available yet, prognoses for individuals diagnosed with pulmonary fibrosis is poor and the median survival of patients with idiopathic pulmonary fibrosis is only 3–5 years. Estimated mortality rates are 64.3 deaths per million in men and 58.4 deaths per million in women (Ryerson and Kolb 2018). A wide range of environmental and occupational inhalation hazards like beryllium, nylon flock, polyvinyl chloride, carbon nanotubes, asbestos or silica are known to engender pulmonary fibrosis (Boag et al. 1999; Cordasco et al. 1980; Dong and Ma 2016; Mossman and Churg 1998; Newman et al. 1996; Vehmas et al. 2012; Yoshida et al. 2011). Likewise cigarette smoke and cancer chemo- or radiotherapy have also been linked to this devastating disease (Giuranno et al. 2019; Morse and Rosas 2014; Sleijfer 2001). Noteworthy, in light of the ongoing Covid-19 pandemic, it is a matter of debate as to whether pulmonary fibrosis treatment might ameliorate the development of severe Covid-19 cases and that pulmonary fibrosis might occur as a long-term consequence of the Covid-19 acute respiratory distress syndrome (George et al. 2020; Ojo et al. 2020; Vasarmidi et al. 2020). Therefore, the incidence of pulmonary fibrosis is expected to further increase in the years to come and thus, the demand for effective therapy options will continue to rise.

Pulmonary fibroblasts are recognized as the major source of extracellular ECM proteins like collagens and fibronectin (Pardo and Selman 2016; Peyser et al. 2019). Innate immune cells such as macrophages and platelets release TGF-β1 and thereby induce migration of activated fibroblasts to the injured lung epithelium (Jiang et al. 2014). Initially, this process leads to beneficial ECM secretion and wound healing (Wilson and Wynn 2009). However, sustained activation of fibroblasts by TGF-β1 results in fibroblast-to-myofibroblast transition (FMT) characterized by expression of α-smooth muscle actin (α-SMA) and overproduction of collagens and fibronectin (Habiel and Hogaboam 2017; Lekkerkerker et al. 2012). Hence, TGF-β1-promoted FMT is key in the development and progression of pulmonary fibrosis and cellular mechanisms that regulate ECM production or secretion from fibroblasts became the focus of pulmonary research (Lin et al. 2020).

The fibrinolytic system is a major regulator of the ECM and composed of the proteolytic enzyme plasmin, its precursor plasminogen (Plg) and plasminogen activators (PA) (Deryugina and Quigley 2012). There are two types of PA: urokinase (uPA) and tissue type PA. In wounded tissues plasmin is primarily generated by uPA (Andreasen et al. 2000). Most components of the plasmin system are secreted proteins that act extracellularly. Plg and uPA, however, are bound to the plasma membrane by cognate receptors: PlgR and uPAR (Felez et al. 1991; Miles et al. 1991; Plow et al. 1986, 2012). Thus, active plasmin can be generated either in the extracellular fluid or in close association to the cell membrane (Deryugina and Quigley 2012; Irigoyen et al. 1999). Plasmin has been shown to degrade major components of the ECM such as collagens and fibronectin, suggesting that stimulation of plasmin activity is a promising strategy for the treatment of pulmonary fibrosis (Deryugina and Quigley 2012; Papp et al. 1987; Pins et al. 2000). Activity of PAs is counterbalanced by the product of the *SERPINE1* gene: the plasminogen activator inhibitor type 1 (PAI-1). PAI-1 is also secreted from pHPF and reported to inhibit soluble and cell-associated PA activity (Bharadwaj et al. 2021). Overexpression of *SERPINE1* and concomitant decreased plasmin activity is strongly associated with pulmonary fibrosis (Ghosh and Vaughan 2012; Huang et al. 2012; Lin et al. 2020; Shioya et al. 2018; Zhang et al. 2012).

TGF-β1 activates transmembrane receptors of the serine/threonine kinase receptor family, which phosphorylate SMAD proteins and trigger their translocation into the nucleus where they induce SMAD-responsive genes in pulmonary fibroblasts (Heldin and Moustakas 2016). This process enhances ECM deposition via two pathways. First, SMADs directly induce transcription of fibronectin/collagens and thus promote ECM production (Vindevoghel et al. 1998; Zhao et al. 2002). Second, SMADs inhibit ECM degradation by activation of the *SERPINE1* gene and concomitant reduction of plasmin activity (Hua et al. 1999; Song et al. 1998). Thus, TGF-β1 is a key player in the propagation of pulmonary fibrosis and inhibition of TGF-β1 signaling in pulmonary fibroblasts is a promising therapeutic strategy (Fernandez and Eickelberg 2012; Gu et al. 2007; Phan et al. 2005; Saito et al. 2018a, b; Song et al. 1998; Walker et al. 2019). Of note, mitigating TGF-β1-dependent ECM production may be useful to prevent fibrosis or progression at early stages. Additionally, increasing plasmin activity may degrade preformed ECM and thus eventually lead to tissue repair at late stages of the disease (Staab-Weijnitz 2021).

The superfamily of mammalian transient receptor potential (TRP) channels represents a multifunctional group of proteins that consists of 27 members in humans (Wu et al. 2010). In recent years, TRP proteins have attracted much interest as potential drug targets for a wide range of pathological conditions (Koivisto et al. 2022). TRPM7 is a bifunctional protein comprising a cation channel and a serine/threonine kinase moiety which are covalently linked (Monteilh-Zoller et al. 2003; Nadler et al. 2001; Nadolni and Zierler 2018; Ryazanova et al. 2004). TRPM7 is ubiquitously expressed and involved in fundamental cellular processes such as cell survival, proliferation, apoptosis and migration (Fleig and Chubanov 2014; Paravicini et al. 2012). In MRC5 cells, a fetal human lung fibroblast cell line, down-regulation of the TRPM7 protein was shown to decrease TGF-β1-induced collagen and α-SMA synthesis (Yu et al. 2013). However, the molecular and cellular underpinnings of these effects have remained elusive and possible effects of TRPM7 activity on the plasmin system have not been addressed yet. Likewise, it is still unknown whether TRPM7 blockers are able to decrease ECM and/or α-SMA protein levels in pHPF. Interestingly, TRPM7 kinase directly phosphorylates SMAD-2 and enhances acute TGF-β1-induced SMAD-2 activation in isolated T-lymphocytes, offering a potential explanation for the positive effects of TRPM7 on TGF-β1-induced collagen expression observed in MRC5 cells (Romagnani et al. 2017). Furthermore, TRPM7 promotes the development of heart and kidney fibrosis, pointing to a possible role of TRPM7 in signaling pathways leading to fibrotic processes (Du et al. 2010; Rios et al. 2020; Suzuki et al. 2020).

Based on the current literature, we postulated that small-molecule TRPM7 blockers may represent new pulmonary fibrosis therapeutics. To this end, we aimed at identifying a mechanistic link between TRPM7, TGF-β1 and the plasmin system in pHPF. We directly measured plasmin activity using a specific fluorogenic substrate for plasmin and analyzed the effects of two unrelated TRPM7 blockers on untreated and TGF-β1-treated pHPF. We determined protein levels of PAI-1, α-SMA, collagen and fibronectin by Western-blot analysis and Sircol™ assays under identical conditions. Finally, we monitored the effects of TRPM7 blockade on TGF-β1-induced SMAD signaling and *SERPINE1,* fibronectin (*FN1*) and alpha-1 type I collagen (*Col1A1*) mRNA expression.

## Materials and methods

### Chemicals and antibodies

*N*-[(1*R*)-1,2,3,4-Tetrahydro-1-naphthalenyl]-1*H*-benzimidazol-2-amine hydrochloride (NS-8593), apamin, human transforming growth factor (TGF-β1; T7039), plasminogen, α2-antiplasmin and D-Val-Leu-Lys-7-amido-4-methylcoumarin were all from SigmaAldrich. Waixenicin A was isolated as described previously (Zierler et al. 2011). For protein detection specific antibodies against PAI-1 (abcam, ab66705), fibronectin (abcam, ab2413), smooth muscle actin (abcam, ab5694), collagen-1 (abcam, ab34710), p-SMAD-2 (cell signaling, clone 138D4, #3108), SMAD-3 (cell signaling, clone C67H9, #9523), SMAD-2 (cell signaling, clone D43B4, #5339), SDHA (abcam, ab14715) and histone H3 (abcam, ab1791) were used.

### Cell culture

pHPF were purchased from PromoCell (C-12360) and cultured using fibroblast growth medium II provided by the manufacturer. Data obtained with cells from two different donors were combined (a 79 and a 44-year-old female). Cells were used between passage 2 and 10. In Fig. 2d and Suppl. Fig. S1, data obtained with pHPF from Lonza (CC-2512) are shown. These cells were also cultured in fibroblast growth medium II provided by the manufacturer and derived from a male donor 37 years of age.

### Stimulation procedure

**Fig. 1:**
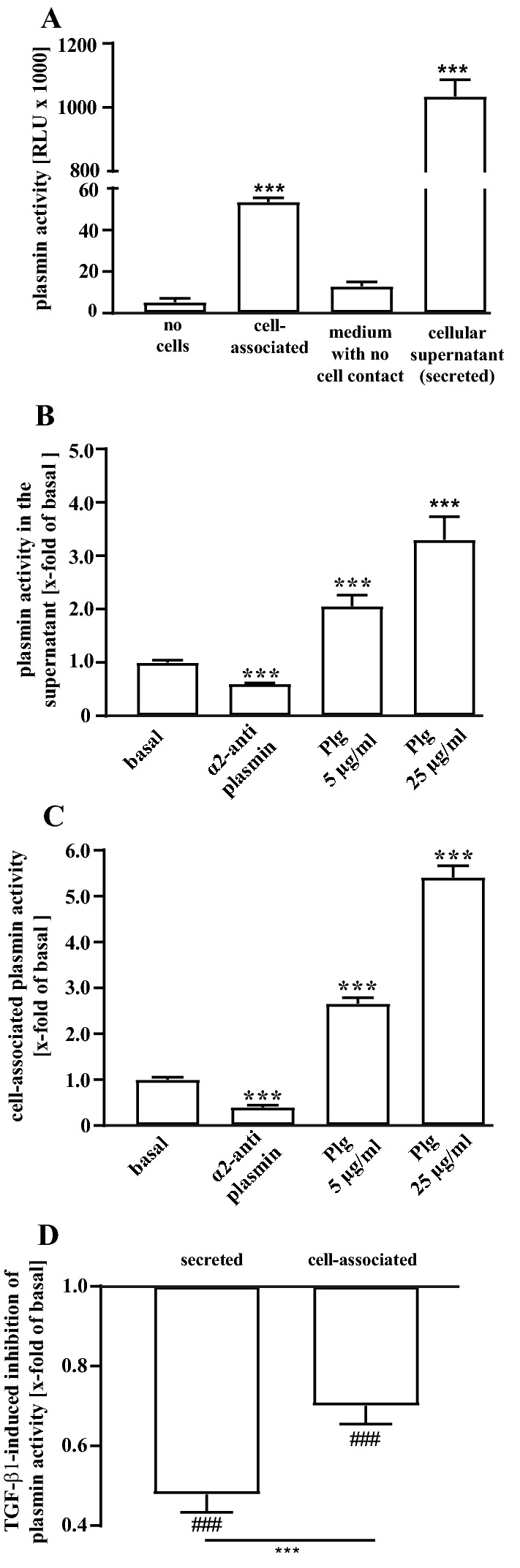
Detection of plasmin activity in living pHPF by D-Val-Leu-Lys-AMC. **a** D-Val-Leu-Lys-AMC (50 µM) was incubated with unstimulated pHPF, without any cells or with fresh medium for 3 h at 37 °C and secreted and cell-associated fluorescence measured. Bars represent SEM of RLU, *n* = 10. **b** secreted and **c** cell-associated plasmin activity was measured after incubating the cells with Plg (5 or 25 µg/ml) for 24 h at 37 °C. α_2_-antiplasmin (500 nM) was co-administrated with D-Val-Leu-Lys-AMC. Bars represent SEM of RLU, *n* = 4. **d** Plasmin activity was measured after stimulation of the cells with TGF-β1 (2 ng/ml) for 24 h. Bars represent SEM of x-fold of basal values, *n* = 10. Statistical analysis was performed using one‐way ANOVA (**a**–**c**) followed by Tukey’s post-test or one- and two-sample *t* test (in **c**) using the GraphPad prism software 9.1. Asterisks indicate in **a** significant differences to “no cells”, in **b** and **c** to basal and in **d** between the cellular fractions. Hash signs indicate significant differences to 1.0

**Fig. 2:**
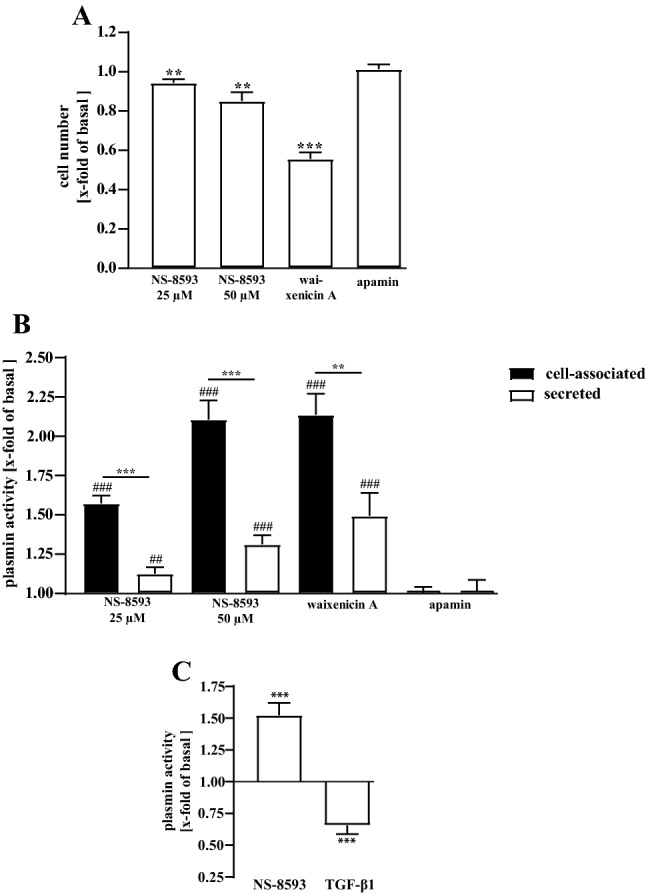
Detection of plasmin activity in living pHPF. **a** pHPF were stimulated with NS-8593 (25 or 50 µM), with waixenicin A (10 µM) or apamin (100 nM) for 24 h and cell numbers determined using SRB. For NS-8593 corresponding DMSO and for waixenicin A ethanol controls were used. Bars represent SEM of x-fold of basal (DMSO or ethanol) values, n = 4–6. **b** pHPF were stimulated with NS-8593 (25 or 50 µM), with waixenicin A (10 µM) or apamin (100 nM) for 24 h and cell-associated and secreted plasmin activity determined. For NS-8593 corresponding DMSO and for waixenicin A ethanol control were used. Bars represent SEM of x-fold of basal values, *n* = 4–6. **c** Cell-associated plasmin activity was measured after stimulation of the cells with TGF-β1 (2 ng/ml) or NS-8593 (25 µM) for 24 h. Bars represent SEM of x-fold of basal values, *n* = 4. In **b**, data obtained with two distinct donors provided from PromoCell (C-12360) and in **c**, from one donor provided by Lonza (CC-2512) are shown. Statistical analysis was performed using one-sample *t* test or one‐way ANOVA followed by Tukey´s post-test using the GraphPad prism software 9.1. Asterisks indicate in **b** significant differences between the cellular fractions and in **a** and **c** to 1.0. In **b** hash signs indicate significant differences to 1.0

**Fig. 3:**
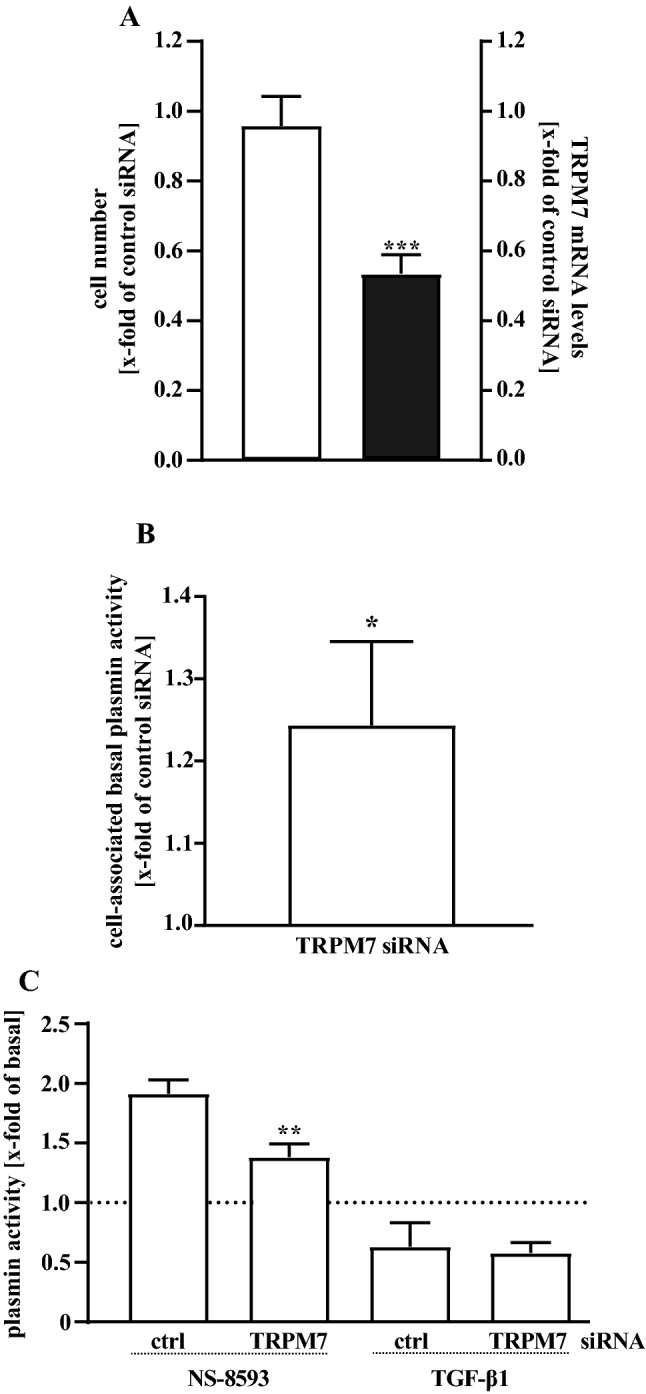
Detection of plasmin activity in living pHPF. pHPF were transfected with a pool of two distinct siRNAs against TRPM7 or a random control siRNA. In **a** 72 h post transfection of TRPM7 specific or control siRNAs cell numbers were determined using SRB (left *y*-axes) or TRPM7 mRNA detected by qRT-PCR (right *y*-axes). In **b** plasmin activity of unstimulated cells was determined. Bars represent SEM of x-fold of the control siRNA, *n* = 4. **c** 48 h post transfection pHPF were stimulated with TGF-β (2 ng/ml) or NS-8593 (25 µM) for 24 h and secreted (TGF-β1) or cell-associated (NS-8593) plasmin activity determined. Bars represent SEM of x-fold of basal values, *n* = 4. Statistical analysis was performed using one-sample *t* test or one‐way ANOVA followed by Tukey s post-test using the GraphPad prism software 9.1. Asterisks indicate in **a** and **b** significant differences to 1.0 and in **c** between control and TRPM7 siRNA

**Fig. 4:**
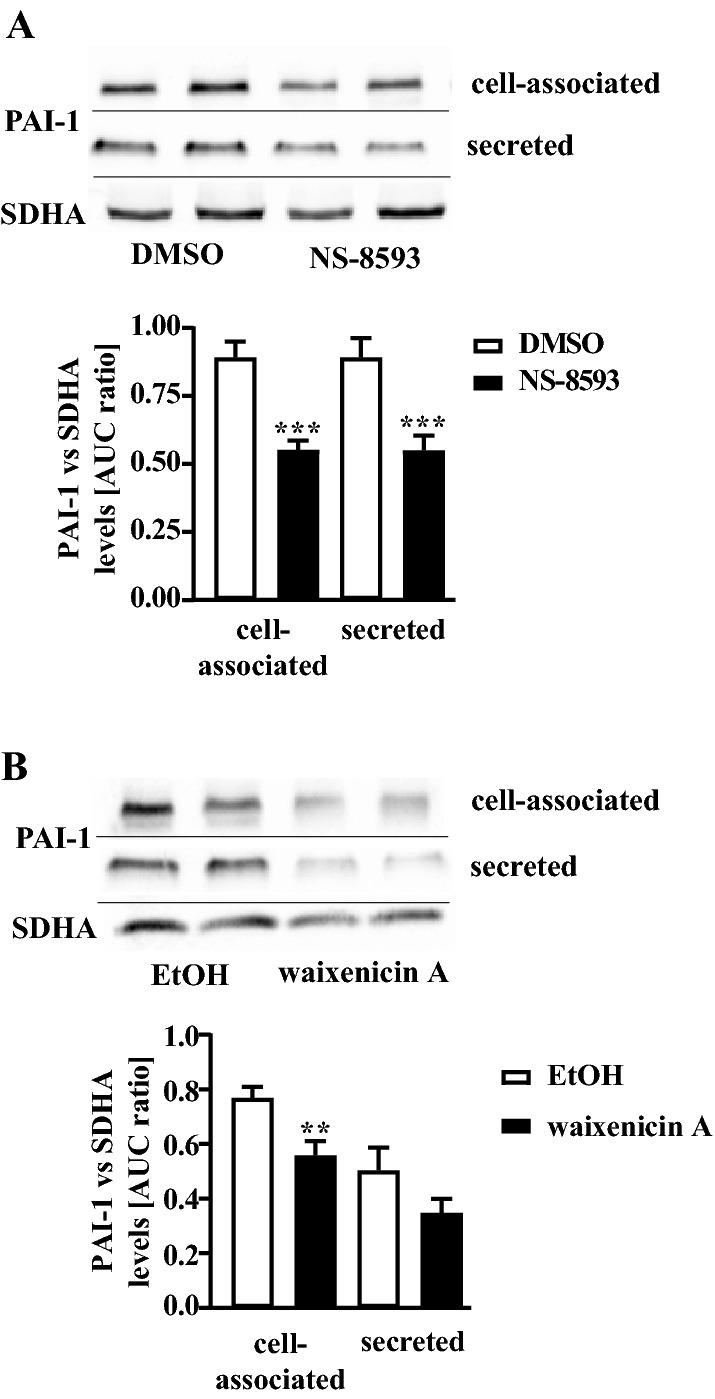
Detection of PAI-1 protein levels in pHPF. **a** cells were stimulated with NS-8593 (25 µM) and** b** with waixenicin A (10 µM) for 24 h and protein amount of PAI-1 or SDHA (loading control) determined. SDHA control of the cellular fraction was also used for the secreted fraction. Blots of the cellular fraction were cut in half and the upper part used for detection of PAI-1 and the lower part for SDHA. Resulting signals were quantified by densitometry and AUC ratios between PAI-1 and SDHA calculated. One set of representative blots is shown. Bars represent SEM of % AUC ratios, *n* = 4. Statistical analysis was performed using one‐way ANOVA followed by Tukey’s post-test using the GraphPad prism software 9.1. Asterisks indicate significant differences between basal (DMSO or EtOH) and the TRPM7 blocker

**Fig. 5:**
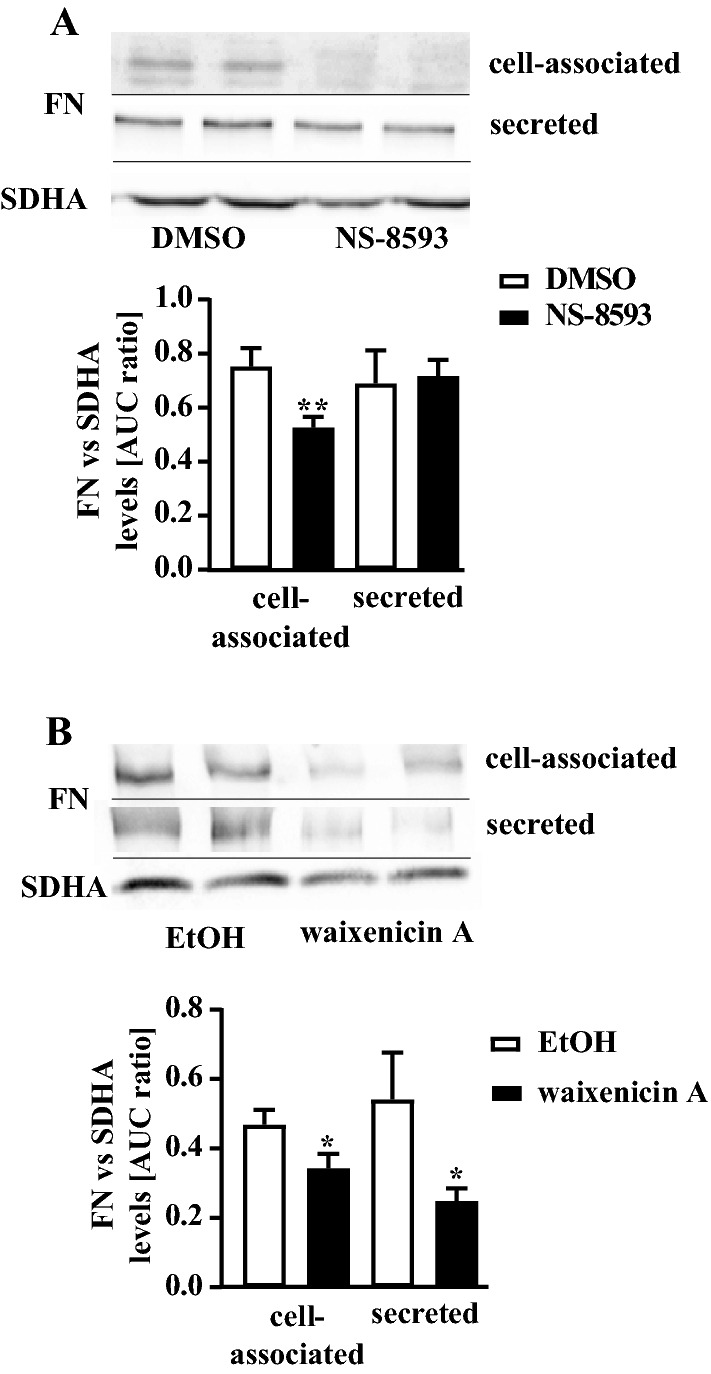
Detection of fibronectin (FN) protein levels in pHPF. **a** cells were stimulated with NS-8593 (25 µM) and** b** with waixenicin A (10 µM) for 24 h and protein amount of FN or SDHA (loading control) determined. SDHA control of the cellular fraction was also used for the secreted fraction. Blots of the cellular fraction were cut in half and the upper part used for detection of FN and the lower part for SDHA. Resulting signals were quantified by densitometry and AUC ratios between FN and SDHA calculated. One set of representative blots is shown. Bars represent SEM of % AUC ratios, *n* = 3. Statistical analysis was performed using one‐way ANOVA followed by Tukey’s post-test using the GraphPad prism software 9.1. Asterisks indicate significant differences between basal (DMSO or EtOH) and the TRPM7 blocker

**Fig. 6:**
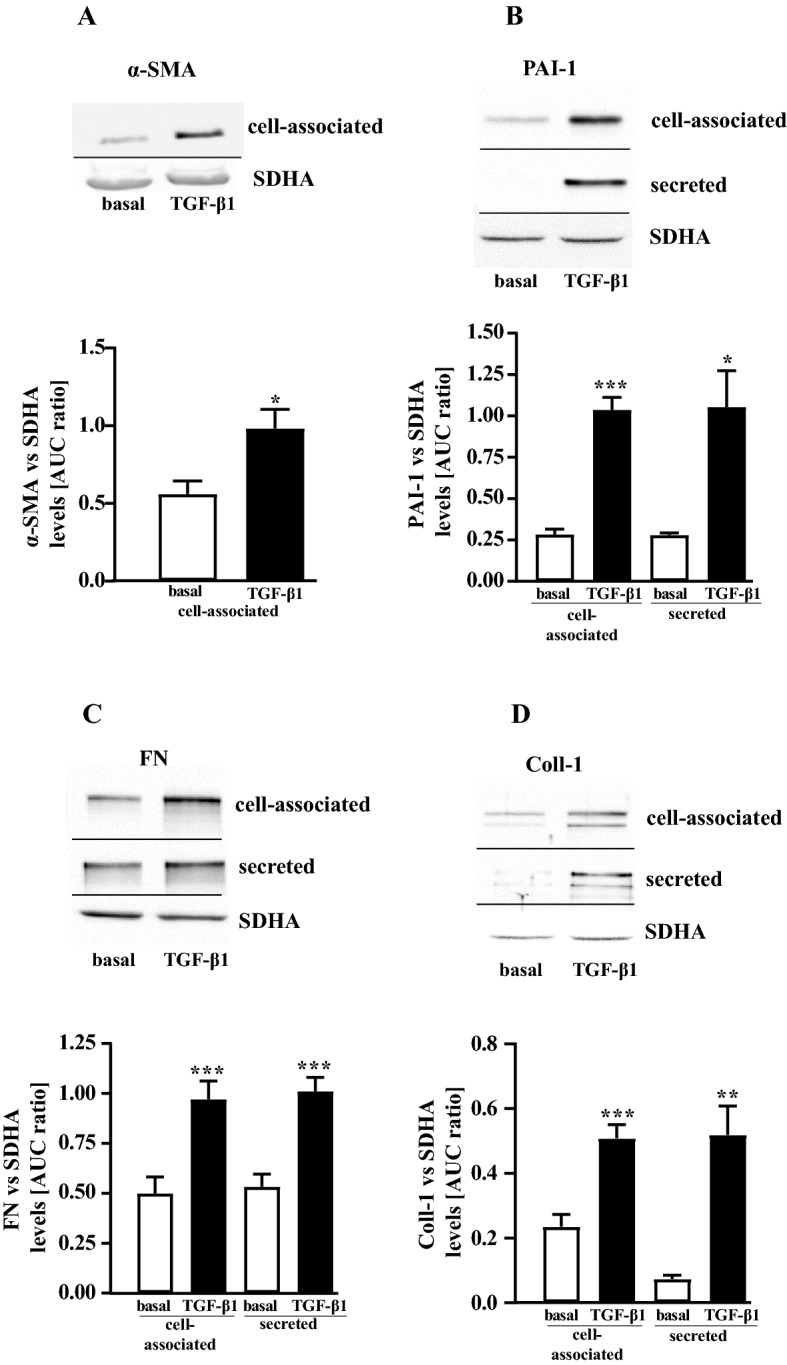
Detection of FMT markers in pHPF treated with TGF-β1. pHPF were stimulated with TGF-β1 (2 ng/ml) for 48 h. Protein amount of α-SMA in **a,** of PAI-1 in **b,** of FN in **c** and of collagen-1 (Coll-1) in **d** was determined in the cell-associated and secreted fraction by western blotting. SDHA control of the cellular fraction was also used for the secreted fraction. Blots of the cellular fraction were cut in half and the upper part used for detection of α-SMA, PAI-1, FN or Coll-1 and the lower part for SDHA. Resulting signals were quantified by densitometry and AUC ratios of α-SMA, PAI-1, FN or Coll-1 and SDHA calculated. One set of representative blots is shown. Bars represent SEM of % AUC ratios, *n* = 3–5. Statistical analysis was performed using one‐way ANOVA followed by Tukey’s post-test using the GraphPad prism software 9.1. Asterisks indicate significant differences between basal and TGF-β1

**Fig. 7:**
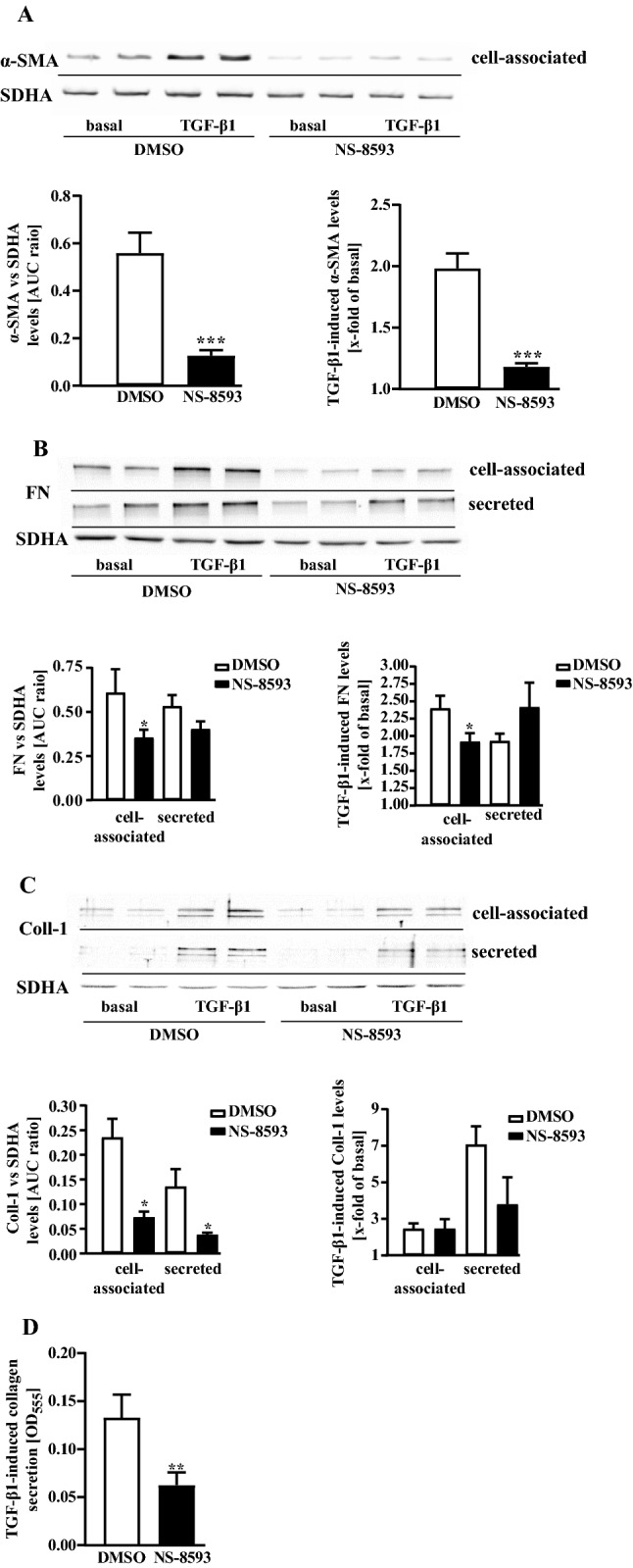
Detection of FMT markers in pHPF co-treated with TGF-β and NS-8593. pHPF were stimulated with TGF-β1 (2 ng/ml) or NS-8593 (25 µM) for 48 h alone or together with both ligands. Protein amount of α-SMA in **a,** of FN in **b** and of collagen-1 (Coll-1) in **c** was determined in the cell-associated and secreted fraction by western blotting. SDHA control of the cellular fraction was also used for the secreted fraction. Blots of the cellular fraction were cut in half and the upper part used for detection of α-SMA, FN or Coll-1 and the lower part for SDHA. Resulting signals were quantified by densitometry and AUC ratios of α-SMA, FN or Coll-1 and SDHA calculated. One set of representative blots is shown. Bars represent SEM of % AUC ratios (NS-8593) or x-fold of basal values (TGF-β1), *n* = 3–5. In **d**, pHPF were stimulated with TGF-β1 (2 ng/ml) alone or together with NS-8593 (25 µM) for 48 h and secreted collagen levels determined by Sircol™ soluble collagen assay. Bars represent SEM of OD_555_ values, *n* = 5. Statistical analysis was performed using one‐way ANOVA followed by Tukey’s post-test using the GraphPad prism software 9.1. Asterisks indicate significant differences between DMSO and NS-8593

**Fig. 8:**
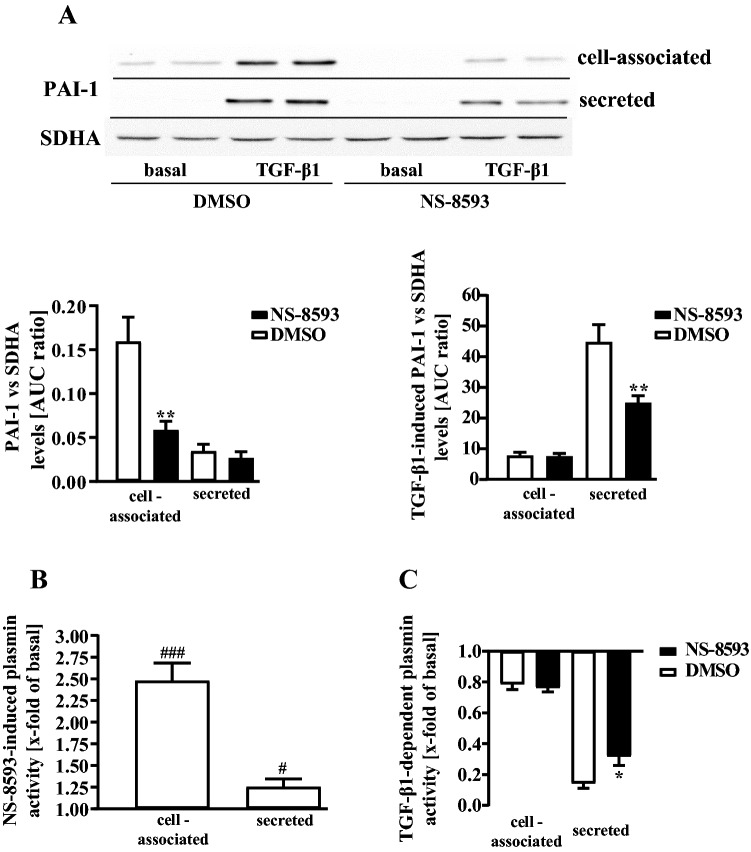
Detection of PAI-1 protein levels and plasmin activity in pHPF co-treated with TGF-β1 and NS-8593. **a** pHPF were stimulated with TGF-β1 (2 ng/ml) or NS-8593 (25 µM) for 48 h alone or together with both ligands. Protein amount of PAI-1 in the cell-associated and secreted fraction was determined by western blotting. SDHA control of the cellular fraction was also used for the secreted fraction. Blots of the cellular fraction were cut in half and the upper part used for detection of PAI-1 and the lower part for SDHA. Resulting signals were quantified by densitometry and AUC ratios of PAI-1 and SDHA calculated. One set of representative blots is shown. Bars represent SEM of PAI-1/SDHA expression ratios, *n* = 3–5. Asterisks indicate significant differences between DMSO and NS-8593, hash signs between basal and TGF-β1. In **b**, pHPF were stimulated with NS-8593 (25 µM) alone and in **c** only with TGF-β1 (2 ng/ml) or together with NS-8593 (25 µM) for 48 h and plasmin activity determined. In **b**, bars represent SEM of x-fold of basal (DMSO), *n* = 5. In **c**, bars represent SEM of x-fold of basal values, *n* = 5. Statistical analysis was performed using one-sample *t* test or one‐way ANOVA followed by Tukey’s post-test using the GraphPad prism software 9.1. In **a**, asterisks indicate significant differences between DMSO and NS-8593 in the absence of TGF-β and hash signs in the presence of TGF-β. In **b**, hash signs indicate significant differences to 1.0. In **c**, asterisks indicate significant differences between DMSO and NS-8593

**Fig. 9:**
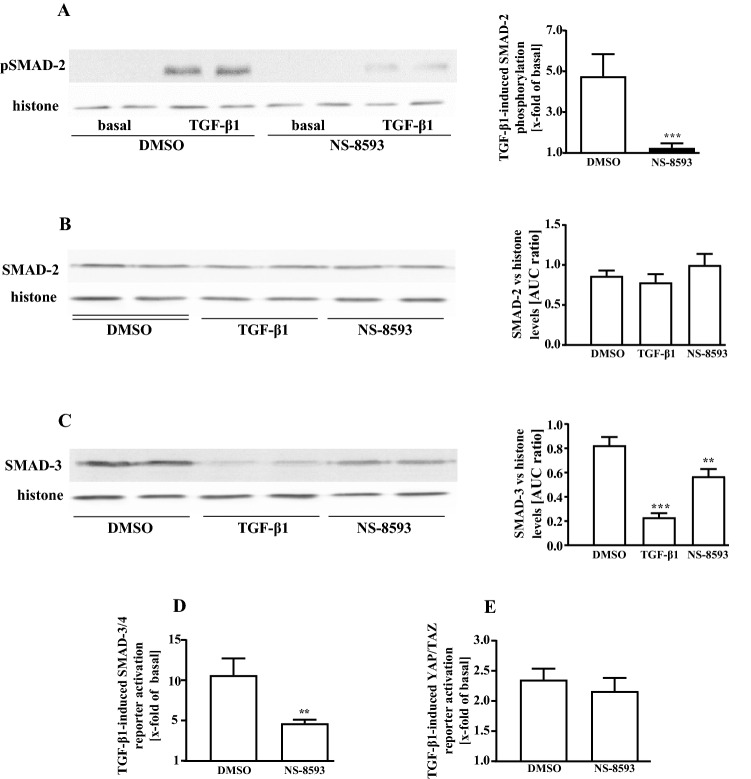
NS-8593 inhibits TGF-β1-induced SMAD activation in pHPF. pHPF were stimulated with TGF-β1 (2 ng/ml) or NS-8593 (25 µM) for 48 h alone or together with both ligands. In **a**, the amount of pSMAD-2, in **b** of total SMAD-2 and in **c** of total SMAD-3 was determined by western blotting. Histone detection served as a loading control. Blots were cut in half and the upper part used for detection of pSMAD-2, SMAD-2 or SMAD-3 and the lower part for histone. Resulting signals were quantified by densitometry and AUC ratios of pSMAD-2, SMAD-2 or SMAD-3 and histone calculated. One set of representative blots is shown. Bars represent SEM of % AUC ratios, *n* = 3–8. In **d**, pHPF were electroporated with the pCAGA-luc reporter and in **e** with the 8xGTIIC-luciferase plasmid. After 24 h, cells were stimulated with TGF-β1 (2 ng/ml) alone or together with NS-8593 (25 µM) for 48 h and then luciferase activity determined. Bars represent SEM of x-fold over basal values, *n* = 5. Statistical analysis was performed using one‐way ANOVA followed by Tukey’s post-test using the GraphPad prism software 9.1. Asterisks indicate significant differences to DMSO

**Fig. 10:**
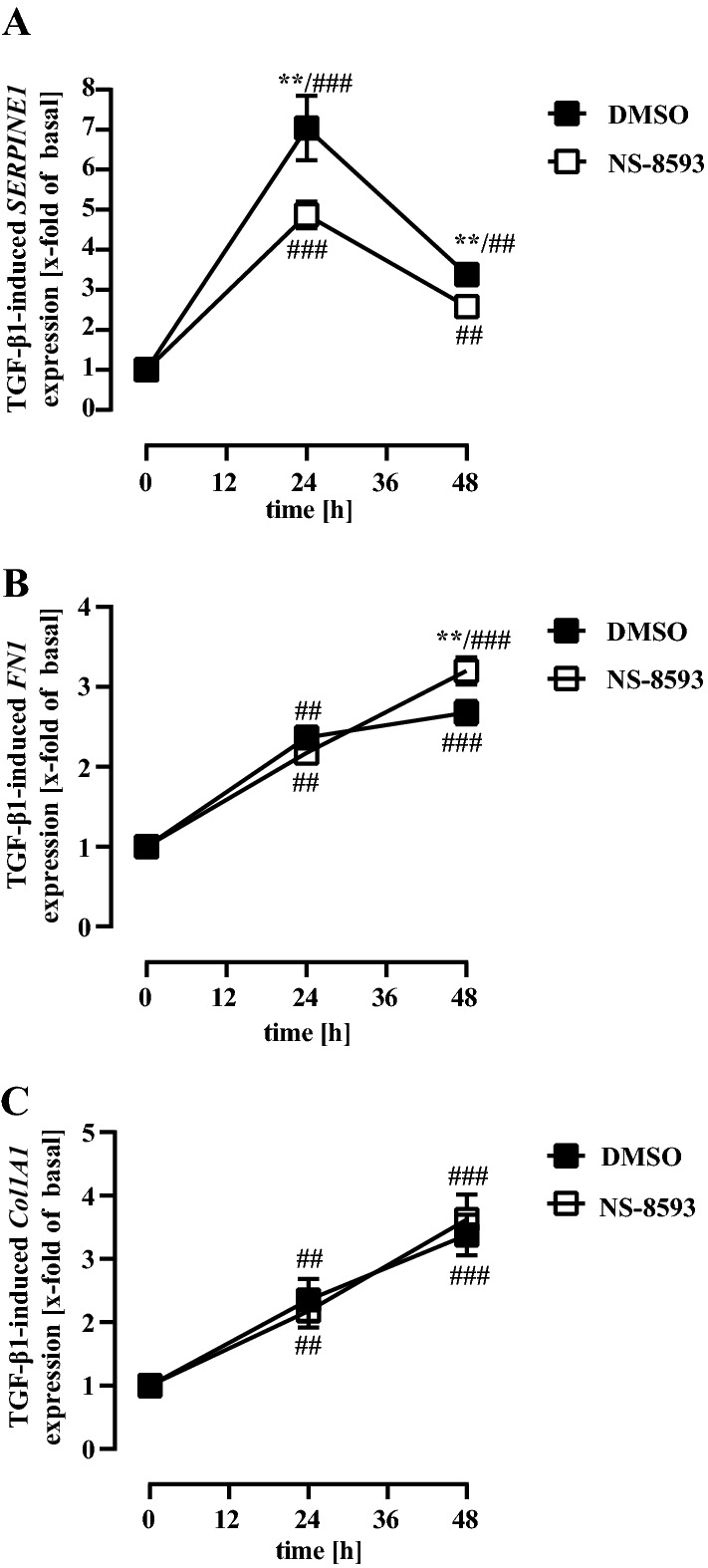
NS-8593 inhibits TGF-β1-induced *SERPINE1* but not *FN1* or *Col1A1* mRNA expression in pHPF. pHPF were stimulated with TGF-β1 (2 ng/ml) for 24 or 48 h alone or together with NS-8593 (25 µM). In **a**, *SERPINE1*, in **b** *FN1* and in **c** *Col1A1* mRNA was determined by qRT-PCR. Data are represented as SEM of x-fold of basal values, *n* = 4. Statistical analysis was performed using two‐way ANOVA followed by Tukey’s post-test using the GraphPad prism software 9.1. Asterisks indicate significant differences to DMSO, hash signs to time point zero

Data shown in Figs. 1–5 were obtained after stimulation of the cells for 24 h in the presence of 5% FCS. In Figs. 6–10, cells were stimulated for 48 h with 0.5% FCS. TGF-β1 treatment of pHPF for 48 h with reduced FCS levels has been established to induce FMT (Malmstrom et al. 2004; Staab-Weijnitz et al. 2015; Thannickal et al. 2003). NS-8593 was dissolved in DMSO (50 mM final), waixenicin A (1 mM) in ethanol and plasminogen (25 mg/ml final) in glycerol. Thus, in each experiment appropriate carrier controls were used. Apamin and TGF-β1 were dissolved in water.

### Plasmin activity

D-Val-Leu-Lys-7-amido-4-methylcoumarin (D-Val-Leu-Lys-AMC) was used as a plasmin substrate (Gyzander and Teger-Nilsson 1980; Kato et al. 1980; Li et al. 2018; Schuliga et al. 2011; Wu et al. 2019). D-Val-Leu-Lys-AMC is a selective fluorogenic substrate for plasmin and enzymatic activity is quantified by release of the free AMC fluorophore, which is excited at 360–380 nm and emits light at 440–460 nm. In detail, ~ 5,000 pHPF were seeded per cavity of a 96-well plate 24 h before the experiment. Cells were stimulated or not with 50 µl medium for the indicated periods of time. Because active plasmin remains first associated to the cell surface and is then subsequently secreted (Deryugina and Quigley 2012), we measured plasmin activity in two cell fractions. To detect secreted plasmin activity, 10 µl of the supernatant was transferred to a new 96-well plate and added to 90 µl Tris/HCl (20 mM, pH 7.4) containing 55 µM of D-Val-Leu-Lys-AMC. For detection of cell and cell-associated plasmin activity, the remaining cell culture medium was aspirated and 50 µL Tris/HCl (20 mM, pH 7.4) with 50 µM D-Val-Leu-Lys-AMC directly added to the cells. After 3 h incubation at 37 °C, fluorescence was measured using a FLUOstar® Omega plate reader. To normalize fluorescence signals to the total protein amount, a second 96-well plate was prepared, treated equally and protein amount monitored by sulforhodamine B (SRB) colorimetric assay (Vichai and Kirtikara 2006). SRB signals were calibrated to the cell number. Plasmin activity was determined as the ratio of the RLU values of the D-Val-Leu-Lys-AMC detection and the OD values of the SRB measurement.

### TRPM7 down-regulation with siRNA

To down-regulate *TRPM7* expression, two pre-designed siRNAs from Ambion (AM16708: ID103360 and ID104677) were incorporated into pHPF by Lipofectamine RNAiMAX Transfection Reagent (#13778100, Thermo Fisher), and data compared to cells transfected with a control siRNA (AM4611). For each cavity of a 96-well plate, 1 pmol siRNA was mixed with 0.3 µl lipofectamine in Opti-MEM medium (#31985062, Thermo Fisher). After 30 min at RT, 10 µl were added to the bottom of the well. ~ 10,000 cells were added to the siRNA/lipofectamine mixture in growth medium (90 µl). After 3 days in culture, cells were stimulated and plasmin activity measured as described above. As a control, 100,000 cells were seeded on one cavity of a 6-well plate and total RNA extracted after 3 days. TRPM7 mRNA was analyzed as described under *mRNA detection by qRT-PCR*.

### Protein detection by Western blotting

Cells were seeded on 6-well plates (~ 100,000/well), cultured for one day and stimulated for the indicated periods of time. To detect expression of secreted proteins, supernatants were transferred to fresh tubes and lysed with Laemmli buffer (fourfold). The corresponding cell fraction was lysed by directly adding Laemmli buffer (onefold) to the 6-well plates. Lysates were subjected to SDS-PAGE (10%) and proteins transferred to nitrocellulose (Amersham Protran™ 0.45 µm, #10600002) by western blotting. After adding the primary antibody over night at 4 °C, blots were washed and incubated with the corresponding HRP-conjugated secondary antibody (anti-rabbit 1:4000, anti-mouse 1:2000) for 1 h at RT. After intensive washing immune reactivity was detected by monitoring the ECL-dependent light emission with a chemiluminescence detection system (Peqlab, Germany). Resulting signals were quantified by densitometry (ImageJ) and ratios of the protein of interest and the loading control calculated.

### Detection of soluble collagen

 ~ 500,000 cells were seeded on 10-cm dishes 24 h before stimulation of the cells. Afterwards collagen levels were determined using the Sircol™ soluble collagen assay from biocolor in accordance with the manufacturer´s protocol.

### Firefly luciferase reporter gene assay

To monitor SMAD activation, the pCAGA-luc reporter construct containing the SMAD-3/4 sensitive part of the human *SERPINE1* promotor was used (Dennler et al. 1998). Activation of YAP/TAZ was monitored by the 8xGTIIC-luciferase plasmid (#34615) obtained from addgene (Dupont et al. 2011). Plasmids were transfected into pHPF via electroporation using the Neon® transfection system from Invitrogen according to the manufacturer´s protocol. Briefly, for each electroporation step, ~ 250,000 cells together with 5 µg of the corresponding plasmid cDNA were challenged 3 times with 1650 V for 10 ms. Cells were immediately placed on 96-well plates (~ 20,000 cell per well) and cultured for 24 h. After stimulation, cells were lysed in 50 µl of lysis buffer (25 mM Tris/HCl pH 7.4, 4 mM EGTA, 8 mM MgCl_2_, 1 mM DTT and 1% Triton-X-100) and a volume of 40 µl transferred to white-bottomed, 96-well plates. Luciferase activity was measured after automatically injecting a luciferase substrate (20 µl) from Promega (E1500) using a FLUOstar® Omega plate reader.

### mRNA detection by qRT-PCR

 ~ 150,000 cells were seeded per cavity of a 6-well plate. After 24 h cells were stimulated or not for the time indicated and stimulation terminated by rapid cooling on ice. Total RNA was isolated using the Trizol® reagent (Invitrogen, Darmstadt, Germany) according to the manufacturer´s instructions. First strand synthesis was carried out with oligo(dT)_18_ primer using 1 µg of total RNA and the RevertAid™ H Minus First Strand cDNA Synthesis Kit (Fermentas, Sankt-Leon Roth, Germany). qRT-PCR was done using the LightCycler® 480 SybrGreen I Master Mix kappa (Roche, Mannheim, Germany). Exon-spanning primer pairs were used at a final concentration of 1 µM each. Final assay volume was 20 µl and first strand synthesis reaction was diluted 1:20 or 1:50. A LightCycler® 480 II (Roche) was used with the following conditions: initial denaturation for 2 min at 94 °C, 55 cycles of 94 °C for 10 s, 55 °C for 10 s and 72 °C for 10 s. Primer design was performed using the ProbeFinder (version 2.53) provided on the website of Roche Life Science. Crossing points (Cp) were determined by the software supplied with the LightCycler® 480 and data analysed by the ΔΔCp method (2^−((gene–*ACTB*)^_TGF-β1/TRPM7 siRNA_^− (gene–*ACTB*))^_basal/control siRNA_). Primer sequences were as follows: *SERPIN1*-forward: 5′-AAGGCACCTCT-GAGAACTTCA-3′, *SERPIN1*-reverse: 5′-CCCAGGACTAGGCAGGTG-3′, *ACTB*-forward: 5′-CTAAGGCCAACCGTGAAAAG-3′, *ACTB*-reverse: 5′-ACCAGAGGCATACAGGGACA-3′, *TRPM7*-forward: 5′-TTGACATTGCCAAAAATCATGT-3′, *TRPM7*-reverse: 5′-CTTGTCCAAGG-ATCCAACC-3′, *FN1*-forward: 5′-CCGACCAGAAGTTTGGGTTCT-3′, *FN1*-reverse: 5′-CAATGC-GGTACATGACCCCT-3′, *COL1A1*-forward: 5′-TACAGAACGGCCTCAGGTACAA-3′, *COL1A1*-reverse: 5′-ACAGATCACGTCATCGCACA-AC-3′.

### Quantification and statistical analysis

Values represent the mean ± SEM of three to eight independent experiments. Statistical analysis was performed using one- or two-sample student’s *t* test, one‐way or two-way ANOVA followed by Tukey’s post-test using the GraphPad prism software 9.1. Shapiro–Wilk tests were performed to ensure normal distribution of the data sets. One symbol indicates a *p*‐value of ≤ 0.05, two of ≤ 0.01 and three of ≤ 0.001.

## Results

### TRPM7 blockers enhance constitutive plasmin activity of pHPF

Despite the importance of the plasmin system for the development of pulmonary fibrosis, plasmin activity has not been systematically investigated directly in pHPF. Hence, we first aimed at establishing a protocol that allows reliable measurements of plasmin activity in cultured pHPF. We used D-Val-Leu-Lys-AMC as a plasmin substrate (Gyzander and Teger-Nilsson 1980; Kato et al. 1980; Li et al. 2018; Wu et al. 2019). D-Val-Leu-Lys-AMC is a selective fluorogenic substrate for plasmin, and enzymatic activity is quantified by the release of the free AMC fluorophore. Indeed, when D-Val-Leu-Lys-AMC was incubated either with an aliquot of pHPF supernatant (secreted) or directly with cells (cell-associated), fluorescence signals were profoundly increased compared to the same amount of substrate, which had not been in contact with any cell fraction (Fig. 1a). Further, medium, which had never been in contact with cells, showed no significant fluorescence increase, indicating that plasmin activity in the secreted fraction was indeed produced by pHPF (Fig. 1a). To test whether this increase in fluorescence could be attributed to plasmin activity, we analyzed effects of the plasmin inhibitor α2-antiplasmin or the plasmin precursor Plg (Moroi and Aoki 1976). As indicated in Fig. 1b, c, the plasmin inhibitor significantly decreased fluorescence signals generated by D-Val-Leu-Lys-AMC and Plg enhanced these signals in the secreted and the cell-associated fraction. Thus, in both fractions there was a clear-cut correlation between D-Val-Leu-Lys-AMC-based fluorescence and plasmin activity. Based on the enhancing effects of TGF-β1 on PAI-1 protein levels, it is expected that TGF-β1 decreases plasmin activity (Horowitz et al. 2008; Schuliga et al. 2011; Song et al. 1998). Thus, to finally validate whether this assay would be able to monitor the dynamic regulation of plasmin activity in pHPF, we treated cells for 24 h with TGF-β1. In previous studies using airway smooth muscle cells or IMR-90 fibroblasts, exogenous Plg was added to increase basal plasmin activity and thus to detect the inhibitory effects of TGF-β1 (Horowitz et al. 2008; Schuliga et al. 2011). As we found very robust basal signals without adding exogenous Plg to pHPF (Fig. 1a), we did not use additional Plg. As expected, TGF-β1 reduced D-Val-Leu-Lys-AMC-dependent fluorescence to 0.48 ± 0.04 fold of basal in the secreted and to 0.7 ± 0.05 in the cell-associated fraction (Fig. 1d), indicating that this protocol reliably monitors plasmin activity of living pHPF.

NS-8593 has been identified as a small-molecule TRPM7 blocker (Chubanov and Gudermann 2020). Due to the fundamental role of TRPM7 in cell homeostasis, prolonged inhibition of TRPM7 activity has been proposed to induce some cell death under certain circumstances (Chubanov et al. 2012). Thus, we first tested how pHPF treatment with 25 or 50 µM of NS-8593 for 24 h affects cell numbers. As shown in Fig. 2a, 25 µM of the TRPM7 blocker reduced the number of pHPF to 0.94 ± 0.02 fold of basal and 50 µM to 0.85 ± 0.04. Thus, overall cytotoxicity of NS-8593 was rather moderate. Next, we asked whether there is a possible link between TRPM7 and plasmin activity. To this end, we treated cells with both NS-8593 concentrations for 24 h, measured plasmin activity in both fractions and normalized the data to the cell number. An increase in plasmin activity of 2.1 ± 0.6 fold of basal was found in the cell-associated and of 1.3 ± 0.6 in the secreted fraction for 50 µM and of 1.6 ± 0.3 and 1.2 ± 0.2, respectively, for 25 µM NS-8593 (Fig. 2b). NS-8593 blocks TRPM7 but also SK potassium (KCa2.1–2.3) channels (Chubanov et al. 2012). Therefore, we used the selective KCa2.1–2.3 blocker apamin as a control (Lamy et al. 2010). Apamin had no effects at all on plasmin activity (Fig. 2b), strongly suggesting that TRPM7 and not KCa2.1–2.3 channels affect the plasmin system in pHPF. To support this notion, we used a second structurally unrelated TRPM7 blocker. Waixenicin A, a xenicane diterpenoid from the Hawaiian soft coral *Sarcothelia edmondsoni* has also been reported to block TRPM7 activity (Zierler et al. 2011). 10 µM waixenicin A reduced the number of pHPF to 0.56 ± 0.03 fold of basal, indicating significant higher cytotoxicity of waixenicin A compared to NS-8593 (Fig. 2a). However, Waixenicin A increased, when normalized to the cell number, cell-associated plasmin activity to 2.2 ± 0.1 and secreted activity to 1.5 ± 0.2 fold of basal (Fig. 2b). Thus, it appeared that TRPM7 activity restrains plasmin activity in pHPF. Accordingly, two structurally unrelated TRPM7 blockers reverse this process and enhance plasmin activity.

Data presented so far were obtained using pHPF derived from two distinct donors provided by the same supplier. We next used cells from a third donor and an independent second provider, to clarify whether the observed effects of TRPM7 blockade represent a common feature of cultured pHPF. As shown in Fig. 2c, a significant increase in plasmin activity (1.5 ± 0.1 of basal) induced by NS-8593 (25 µM) was also detectable with cells from the third donor, indicating that the functional link between TRPM7 blockers and plasmin activity is not dependent on the origin of fibroblasts. In line with this notion, TGF-β1-induced reduction in plasmin activity was also very similar in all cell pools (Figs. 1a, 2c). Finally, we aimed at defining the target of NS-8593 in pHPF. To this end, we introduced human TRPM7-specific siRNAs into pHPF and compared resulting cell numbers and TRPM7 mRNA levels to cells transfected with a control siRNA. As shown in Fig. 3a, TRPM7 siRNAs did not significantly affect cell numbers of pHPF but decreased TRPM7 mRNA levels to 0.53 ± 0.05 fold of basal TRPM7 levels detected in the presence of the control siRNA. TRPM7 siRNA-induced reduction of TRPM7 mRNA was accompanied with slightly increased basal plasmin activity (Fig. 3b), further substantiating the notion that TRPM7 restrains plasmin activity in pHPF. Furthermore, effects of NS-8593 but not of TGF-β1 on plasmin activity were significantly reduced in cells transfected with TRPM7 specific siRNAs (Fig. 3c), reinforcing the concept that the TRPM7 protein is the cellular target that links NS-8593 to the plasmin system.

### TRPM7 blockers decrease PAI-1 and fibronectin protein levels in pHPF

Dynamic regulation of the *SERPINE1* gene leads to altered PAI-1 protein levels and is a common plasmin activity regulating mechanism in pHPF (Ghosh and Vaughan 2012). Thus, we postulated that enhanced plasmin activity following TRPM7 blockade might be associated with reduced PAI-1 protein levels. Western-blot data shown in Fig. 4 clearly indicate that incubation of pHPF with NS-8593 (25 µM) or waixenicin A (10 µM) for 24 h inhibited basal PAI-1 levels in the cell-associated and secreted fraction. Next, we tested whether levels of fibrosis-relevant ECM proteins such as fibronectin correlate with the observed increase in plasmin activity ensuing TRPM7 blockade. Both blockers significantly reduced fibronectin levels in the cell-associated fraction (Fig. 5a, b). Hence, we identified TRPM7 blockers as new experimental tools for the regulation of plasmin activity and fibronectin levels of untreated, inactive pHPF.

### TRPM7 blockade inhibits TGF-β1-induced fibroblast-to-myofibroblast transition

TGF-β1-treated, activated pHPF undergo a transition to myofibroblasts characterized by increased protein levels of α-SMA and PAI-1. Further, myofibroblasts secrete high amounts of ECM proteins such as collagens and fibronectin (Habiel and Hogaboam 2017; Lekkerkerker et al. 2012). When cultured pHPF were stimulated with TGF-β1 for 48 h under reduced serum concentrations (0.5%), the entire set of FMT markers was detectable. α-SMA levels increased within the cells (Fig. 6a) and the amount of PAI-1, fibronectin and collagen-1 proteins robustly rose in the cell-associated and secreted fraction (Fig. 6b–d). Next, we wondered whether TRPM7 blockade would counteract FMT. Thus, we left pHPF untreated or challenged them with NS-8593 (25 µM) or TGF-β1 alone or with both ligands for 48 h. The TRPM7 blocker significantly reduced α-SMA levels in untreated and TGF-β1-treated cells (Fig. 7a), suggesting that TRPM7 inhibition prevents FMT. In line with this notion, NS-8593 reduced basal and TGF-β1-promoted fibronectin levels in the cell-associated fraction (Fig. 7b) and strongly reduced the amount of collagen-1 in both fractions under basal conditions (Fig. 7c). However, in TGF-β1-treated cells no significant effect of NS-8593 on collagen-1 secretion was observed, despite a strong tendency of the TRPM7 blocker to inhibit TGF-β1-induced collagen-1 levels in the supernatant. Hence, we used a second approach (Sircol™ soluble collagen assay) to detect collagens in pHPF cultures. Data obtained with the Sircol™ assay revealed highly significant inhibition of TGF-β1-induced collagen secretion after TRPM7 blockade (Fig. 7d). Next, we analyzed whether the effects of NS-8593 on TGF-β1 might also occur on the level of the plasmin system. When basal *SERPINE1* expression was analyzed in the cell-associated and secreted fraction by Western blot, no secreted PAI-1 protein was detectable (Fig. 8a). Cell-associated basal PAI-1 levels were significantly inhibited after TRPM7 blockade by ~ 70% (Fig. 8a). TGF-β1-treatment increased PAI-1 levels in the cell-associated and the secreted fraction and co-administration of NS-8593 significantly reduced the amount of PAI-1 protein in the secreted fraction (Fig. 8a). When plasmin activity was monitored, NS-8593 alone enhanced cell-associated enzymatic activity to 2.5 ± 0.2 fold of basal and secreted activity to 1.3 ± 0.1 fold (Fig. 8b). Co-application of NS-8593 elevated TGF-β1-mediated inhibition of plasmin activity in the secreted fraction from 0.15 ± 0.03 fold of basal to 0.32 ± 0.06 (Fig. 8c). Thus, TRPM7 blockade also affected the plasmin system in TGF-β1-treated, activated pHPF.

### TRPM7 blockade inhibits TGF-β1-induced SMAD signaling in pHPF

Phosphorylation of the transcription factor SMAD-2 is crucial for TGF-β1-promoted FMT (Kawarada et al. 2016). Accordingly, treatment of pHPF with TGF-β1 for 48 h induced SMAD-2 phosphorylation (Fig. 9a). In line with its inhibitory effects on TGF-β1-induced FMT, NS-8593 almost completely abolished TGF-β1-promoted phosphorylation of SMAD-2 (Fig. 9a), whereas total SMAD-2 protein levels were unaffected (Fig. 9b). Previous studies revealed that sustained stimulation of MRC5 cells or pHPF with TGF-β1 reduced total protein levels of SMAD-3 (Breton et al. 2018; Staab-Weijnitz et al. 2015). Accordingly, we observed a profound reduction of SMAD-3 levels after TGF-β1 stimulation of pHPF (Fig. 9c). Interestingly, NS-8593 also significantly diminished SMAD-3 levels (Fig. 9c), indicative of hitherto unappreciated positive effects of TRPM7 activity on total SMAD-3 protein levels. Overall, these data indicate functional interactions between TRPM7 activity and TGF-β1-induced SMAD signaling in pHPF. To analyze whether these interactions affect expression of the *SERPINE1* gene, we took advantage of a previously established reporter gene construct containing the SMAD-3/4 sensitive part of the *SERPINE1* promotor (Dennler et al. 1998). As shown in Fig. 9d, NS-8593 strongly inhibited TGF-β1-induced SMAD-dependent reporter activation. TGF-β1 does not only signal via SMAD proteins but also via SMAD-independent activation of transcription factors of the YAP/TAZ family (Miranda et al. 2017). Of note, inhibitory actions of TRPM7 blockade on TGF-β1 signaling were not observed when TGF-β1-induced activation of a YAP/TAZ sensitive reporter was analyzed (Fig. 9e). Finally, we analyzed whether NS-8593-mediated blockade of TGF-β1-induced SMAD signaling is sufficient to inhibit promoter activity of SMAD-dependent genes by monitoring mRNA expression of the *FN1*, the *COL1A1* and the *SERPINE1* gene after 24 and 48 h. TGF-β1 induced expression of all three genes at both time points, but whereas *FN1* and *COL1A1* mRNA levels rose linearly, *SERPINE1* expression peaked after 24 h and sunk afterwards (Fig. 10). Of note, co-treatment with NS-8593 significantly inhibited TGF-β1-induced *SERPINE1* expression at both time points (Fig. 10a) but had no inhibitory effect on *FN1* or *COL1A1* expression (Fig. 10b, c), indicating that absence of TRPM7 activity weakened rather selectively TGF-β1-promoted activation of the *SERPINE1* gene.

## Discussion

Excessive ECM deposition in the lung eventually leads to pulmonary fibrosis and death. TGF-β1-induced SMAD activation enhances ECM deposition by increasing its production and reducing its degradation (Fernandez and Eickelberg 2012; Gu et al. 2007; Phan et al. 2005; Saito et al. 2018a, b; Song et al. 1998; Walker et al. 2019). This process is beneficial for wound healing, but prolongation eventually leads to pathophysiological conditions such as pulmonary fibrosis. Here, we report that activity of the TRPM7 protein supports TGF-β1-promoted ECM deposition on the level of SMAD dependent transcription, *SERPINE-1* expression, plasmin activation and ECM production in pHPF. To the best of our knowledge, this is the first report of a TRP channel involved in the modulation of plasmin activity. Hence, we propose TRPM7 blockers as new tools to modify plasmin activity in pHPF.

Plg and PA act in solution or cell associated when bound to their cognate cell surface receptors (Deryugina and Quigley 2012; Hoyer-Hansen and Lund 2007; Hu et al. 2007; Milenkovic et al. 2017). Therefore, we analyzed the plasmin system in the cell associated and secreted fraction of pHPF. The total amount of secreted basal plasmin activity was ~ 20 times higher compared to the cell-associated fraction. However, it should be noted that the volume of the supernatant (50 µl) by far exceeds the volume of the cellular fraction (~ 0.05–0.1 µl). Hence, when plasmin is released into the supernatant there is a strong dilution effect and the actual plasmin concentration might be even higher in the cell-associated fraction. Despite the outstanding role of TGF-β1-mediated inhibition of plasmin activity for ECM deposition, to the best of our knowledge, direct effects of TGF-β1 on plasmin activity in pHPF have not yet been analyzed. As expected, we found that TGF-β1 decreased plasmin activity in both fractions of pHPF, but stronger in the secreted fraction. Strong effects of TGF-β on secreted plasmin activity are in line with previous studies using other cell models (Horowitz et al. 2008; Schuliga et al. 2011). In contrast to these studies, no exogenous Plg was required to assess the effects of TGF-β1 on plasmin in pHPF, suggesting rather high endogenous Plg levels in these cells. Under these conditions, two unrelated TRPM7 blockers enhanced plasmin activity of pHPF, indicating that TRPM7 activity restrains plasmin activity in pulmonary fibroblasts. In contrast to TGF-β1, NS-8593 elevated plasmin activity largely in the cell-associated fraction. One obvious explanation for this phenomenon could be that activation and inhibition of the plasmin system follows distinct kinetics. Further, effects of NS-8593 on plasmin activity most likely depend on basal PAI-1 levels. As basal cell-associated PAI-1 levels in pHPF were significantly higher than those secreted, it appears reasonable that inhibitory actions on basal PAI-1 levels affected cell-associated plasmin activity stronger than secreted. Finally, the enhancing effects of TGF-β1 on PAI-1 levels were much stronger than the oppressive effects of NS-8593. Smaller changes of basal PAI-1 levels by NS-8593 might not affect PA activity in the supernatant due to the abovementioned dilution effects. However, despite these differential actions, NS-8593 clearly affected TGF-β1-promoted inhibition of the plasmin system. Of note, PAI-1 proteins that are induced and secreted by TGF-β1 have to be produced first in the cell-associated fraction. Hence, inhibitory actions of NS-8593 on the cellular pool of PAI-1 proteins, should consequently lead to reduced TGF-β1-induced PAI-1 secretion. In line with this notion, co-administration of NS-8593 and TGF-β1 significantly reduced cell-associated and secreted PAI-1 levels and enhanced secreted plasmin activity. Furthermore, NS-8593 counteracted TGF-β1-induced production of fibronectin and collagens. Hence, TRPM7 blockade not only has the potential to enhance plasmin activity and thus to degrade ECM in native pHPF, but also in fibroblasts continuously exposed to TGF-β1.

We found a causal relationship between TRPM7 and plasmin activity after 24 and 48 h of channel blockade. Inhibition of TRPM7 activity for 4 h did not affect plasmin activity (data not shown), indicating that acute TRPM7 inhibition is not linked to the plasmin system. Thus, we favor the hypothesis that TRPM7 activity affects plasmin indirectly via modulating gene expression in pHPF. Because TRPM7 blockade decreased TGF-β1-induced SMAD but not YAP/TAZ activation, it is reasonable to assume that SMAD-dependent genes are involved. The *SERPINE1* gene is a SMAD-dependent gene of utmost importance for pulmonary fibrosis (Hua et al. 1999; Song et al. 1998). TRPM7 blockade reduced TGF-β1-induced SMAD-2 phosphorylation, SMAD-3/4 reporter activation and *SERPINE1* expression on the mRNA and protein level. Hence, we provide a substantial body of evidence indicating that TRPM7 supports the SMAD pathway and thereby affects plasmin activity and thus ECM levels via regulation of PAI-1 protein levels. Of note, collagens and fibronectin are also encoded by SMAD-dependent genes (Vindevoghel et al. 1998; Zhao et al. 2002). Thus, lower ECM levels after TRPM7 blockade could also be caused by reduced transcription of these genes. However, TRPM7 blocker did not reduce TGF-β1-induced *FN1* or *Col1A1* mRNA levels. Hence, TRPM7 supports TGF-β1-promoted ECM deposition most likely by depressing plasmin-mediated degradation. These actions might be relevant in late stages of pulmonary fibrosis. Enhanced plasmin activity due to TRPM7 blockade might degrade already deposited ECM, eventually leading to tissue repair or even restoration. Of note, NS-8593 application to mice prevented and ameliorated kidney fibrosis, indicating the effectiveness of TRPM7 blocker in vivo (Suzuki et al. 2020).

At this point, we can only speculate about possible mechanism leading to rather selective effects of TRPM7 on TGF-β1-induced SERPINE1 expression. It appeared that TGF-β1 elevated SERPINE1 mRNA levels with different kinetics compared to *FN1* and *Col1A1,* indicative for distinct mode of actions by the cytokine on SMAD-dependent promoters. Although all three promoters have a regulatory binding site for SMADs in common, they also differ in binding sites for other transcription factors. In line with this notion, TRPM7 blockade selectively affected TGF-β1 signaling leading to SMAD but not to YAP/TAZ activation. Such fine-tuned effects of TRPM7 on TGF-β1 could lead to selective reduction of SERPINE-1 mRNA by TRPM7 blockers. However, it will be an interesting task for future studies to decipher why inhibition of TGF-β1-promoted SMAD activity by TRPM7 blocker affects the *SERPINE1* but not the *FN1* or *Col1A1* promoter.

TRPM7 is a bifunctional protein with an ion channel moiety and a kinase function. The TRPM7 pore is permeable for Mg^2+^, Ca^2+^ and Zn^2+^ ions and the α-kinase domain phosphorylates serine/threonine residues of the TRPM7 protein itself or of other cellular substrates (Koivisto et al. 2022; Monteilh-Zoller et al. 2003; Nadler et al. 2001; Nadolni and Zierler 2018; Ryazanova et al. 2004). Both TRPM7 functions could potentially affect SMAD-dependent gene expression. As no specific TRPM7 blocker is available that would target either the channel or the kinase activity independently from each other, we cannot discriminate between both activities by employing pharmacological tools. Lysine at position 1646 is essential for TRPM7 kinase activity but not for ion channel function (Kaitsuka et al. 2014; Ryazanova et al. 2014). Thus, genetically modified mice models (TRPM7-K1646R mice) have been introduced, in which the TRPM7 wild-type protein was replaced by a lysine 1646 arginine point mutant (Kaitsuka et al. 2014; Romagnani et al. 2017). TRPM7-K1646R mice appeared as useful tools to discriminate TRPM7 kinase from ion channel function in vivo and in vitro. Noteworthy, SMAD-2 is a direct substrate of the TRPM7 kinase and after 10 min of TGF-β1 stimulation SMAD-2 phosphorylation was reduced in isolated T-lymphocytes from TRPM7-K1646R mice compared to wild-type littermates, indicating that TRPM7 kinase activity supports TGF-β1-induced SMAD stimulation in these cells (Romagnani et al. 2017). Hence, we aimed at using isolated pulmonary fibroblasts from TRPM7-K1646R mice, in to analyze a role of the TRPM7 kinase in the regulation of SMAD and plasmin activity in lung fibroblasts. In this process, we soon realized that TRPM7 blockade did not affect TGF-β-induced SMAD-3/4-dependent reporter activation in wild-type mouse fibroblasts (Fig. S1a), indicating species-specific differences in the functional interactions between TRPM7 and the TGF-β system in lung fibroblasts. Furthermore, in contrast to the data obtained with pHPF, TGF-β did not decrease SMAD-3 expression in lung fibroblasts from mice (Fig. S1b), indicating species-specific differences of the TGF-β system independent from TRPM7. Because of these significant species-specific differences of the TGF-β system in pulmonary fibroblasts, we refrained to use mice models to analyze interactions between TRPM7 and TGF-β. Thus, although we provide significant evidence that TRPM7 blockade affects TGF-β signaling at multiple levels in pHPF, at this point, due to the lack of suitable tools, it remains unclear which TRPM7 function is involved.

## Conclusion

The peptidase plasmin plays a central role in the pathophysiology of pulmonary fibrosis, a fatal disease with essentially no treatment. Plasmin activity elevating processes in pHPF very likely ameliorate pulmonary fibrosis. We report that TRPM7 blockers enhance plasmin activity in untreated and TGF-β-treated pHPF. Further, TRPM7 blockade limited SMAD activation and reduced PAI-1, collagen and fibronectin levels. Thus, we identify a so far unappreciated role for the TRPM7 as a positive modulator of the plasmin system in pulmonary fibroblasts and propose TRPM7 blockers as new promising tools to treat pulmonary fibrosis.

## Supplementary Information

Below is the link to the electronic supplementary material.

Supplementary file1 (PDF 663 KB)

## Abbreviations

ECM: Extracellular matrix
FMT: Fibroblast-to-myofibroblast transition
Plg: Plasminogen
PA: Plasminogen activator
PAI-1: Plasminogen activator inhibitor-1
pHPF: Primary human lung fibroblasts
α-SMA: α-Smooth muscle actin
SRB: Sulforhodamine B
TGF-β1: Transforming growth factor-β1
TRP: Transient receptor potential
TRPM7: Transient receptor potential cation channel subfamily M Member 7
