# Supplementary material for: TRPM7 restrains plasmin activity and promotes transforming growth factor-β1 signaling in primary human lung fibroblasts
Source: Arch Toxicol. 2022 Jul 21;96(10):2767–83. doi: 10.1007/s00204-022-03342-x (PMC9302958; doi:10.1007/s00204-022-03342-x)
Supplement: Supplementary file 1 — Supplementary file1 (PDF 663 KB) [file 204_2022_3342_MOESM1_ESM.pdf]

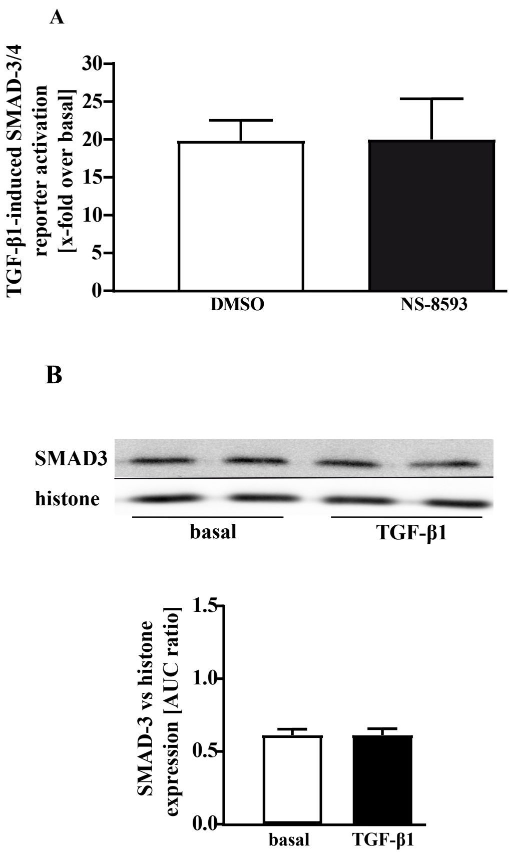

Suppl. Fig. S1 TGF-β signaling in primary pulmonary mouse fibroblasts is distinct from pHPF. Primary pulmonary mouse fibroblasts were isolated as previously described (Staab-Weijnitz et la., Am J Respir Crit Care Med. 2015 Aug 15; 192 (4): 455-67) and electroporated with the pCAGA-luc reporter. After 24 h, cells were stimulated with TGF (2 ng/ml) alone or together with NS-8593 (25 µM) for 48 h and then luciferase activity was determined. Bars represent SEM of x-fold over basal values, n = 3. In **b**, the amount of total SMAD-3 was determined by western-blotting. Histone detection served as a loading control. Blots were cut in half and the upper part used for detection of SMAD-3 and the lower part for histone. Resulting signals were quantified by densitometry and AUC ratios of SMAD-3 and histone calculated. One set of representative blots is shown. Bars represent SEM of % AUC ratios, n = 10.
